# Otitis externa due to allergic contact dermatitis to earphones and earplugs: A case control study

**DOI:** 10.1016/j.jdin.2025.05.020

**Published:** 2025-07-18

**Authors:** Youyou Jin, Wei Hua, Qingfeng Liu, Zili Zheng, Xinyi Yao, Xiangling Zhang, Mei Li, Xiaoyun Zhang, Ran Gao, Xiaoyu Wang, Yueyi Sun, Li Li, Wei Zhang, Chengchen Guo, Yu Zhao, Haotian Liu

**Affiliations:** a Department of Otolaryngology-Head and Neck Surgery, West China Hospital of Sichuan University, Chengdu, China; b Department of Dermatovenereology, West China Hospital of Sichuan University, Chengdu, China; c School of Engineering, Westlake University, Hangzhou, China; d West China Biomedical Big Data Centre, West China Hospital of Sichuan University, Chengdu, China

**Keywords:** allergic contact dermatitis, earphone, earplug, otitis externa, patch test

*To the Editor:* The widespread use of wireless, in-ear headphones and earplugs has been linked to increasing reports of otitis externa (OE) clinical symptoms.^1^, ^2^, ^3^A real-world case-control study on contact dermatitis to in-ear devices was conducted to explore its association with OE and identify potential risk factors for earphone/earplug allergy.

Participants with ear symptoms were included. All participants were tested with a patch test on the back with the 5 common earphone ear tips and earplug samples. A small piece was cut from the ear tip for testing. Testing and reading were performed according to international guidelines.^4^ The patches were applied to the subject’s back for 48 hours, and readings were taken at 48 and 72 hours. The assessors were blinded during the clinical evaluations to minimize bias. Erythematous papules and vesicles with edema were indicative of allergy according to the International Contact Dermatitis Research Group. A questionnaire was used for data collection.

OE patients (*N* = 128) and 50 healthy earphone/earplug users (*N* = 50) were enrolled, with 75 meeting the inclusion and exclusion criteria for patch testing (see Supplementary Table I, available via Mendeley at https://data.mendeley.com/datasets/6zx6hfttfk/1). Participant characteristics are in Table I.

**Table I:** Basic characteristics of OE patients and non-OE volunteers

Variables	Patients (*n* = 38)	Volunteers (*n* = 37)	$x^{2}$/Z/F	*P*
Age (mean ± SD)	25.26 ± 3.998	24.16 ± 3.236	0.853	.195
Women	25 (65.8%)	25 (67.6%)	0.027	.870
Ear symptoms related to earphone/earplug use	38 (100.0%)	34 (91.9%)		.115
Earphone/earplug contact allergy	11 (28.9%)	1 (2.7%)	9.607	.002
Atopic diseases	19 (50.0%)	12 (32.4%)	2.386	.122
Seborrheic dermatitis	26 (68.4%)	25 (67.6%)	0.006	.937
In-ear earphone/earplug	29 (76.3%)	27 (73.0%)	0.111	.739
Material of earphone/earplug				
Resin	11 (28.9%)	12 (32.4%)	0.107	.743
Silicone rubber	24 (63.2%)	21 (56.8%)	0.320	.572
Sponge	16 (42.1%)	13 (35.1%)	0.384	.535
Times of use per wk	10 (7-14)	14 (7-21)	−0.907	.365
Duration of use per d (h)	3 (1.75-5.75)	2 (1.88-4.25)	−0.540	.589
Times of use per d	2 (1-3)	2 (1-3)	−0.516	.606
Volume	40 (30-50)	40 (30-60)	−0.494	.621

Data are presented as mean ± SD or median (IQR) for continuous variables and number (%) for categorical variables.

*P* values were calculated using Pearson's chi-square test for categorical variables and the Mann-Whitney U test for continuous variables, unless otherwise indicated.

*OE*, Otitis externa.

The patch test revealed an overall positivity rate of 16.0% (12/75). Specifically, positive reactions to the ear tips of Galaxy Buds Live were observed in 6 individuals (Fig 1, *A* and *B*). Additionally, 2 participants exhibited doubtful or positive reactions to the ear tips of the AirPods Pro and 3M 1100. Similarly, 3 individuals showed similar reactions to the ear tips of Galaxy Buds2 Pro and Lollipods Pro.

**Fig 1:**
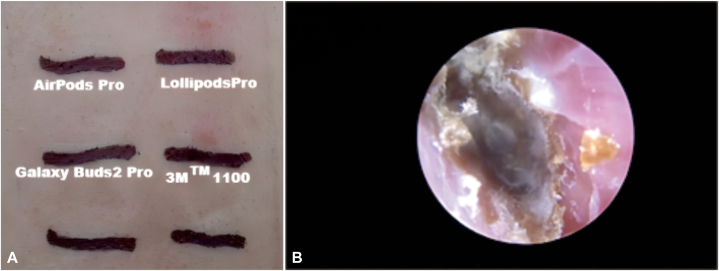
Patch test and fungal findings in patients with otitis externa. **A,** Mild positive (+) patch-test reaction to a Galaxy Buds Live ear tip. **B,** Fungal otitis externa (fungal masses observed under the microscope).

Among individuals with OE, 28.9% (11/38) showed at least 1 positive or doubtful reaction, significantly higher than the 2.7% (1/37) observed in the non-OE group (*P* = .002). Notably, patients with OE exhibited significantly greater reactions to Galaxy Buds Live than non-OE volunteers. However, no significant differences were detected between the 2 groups in the positivity rates of Galaxy Buds2 Pro, Lollipods Pro, AirPods Pro, or 3M 1100.

Analysis of influencing factors showed that individuals with positive responses reported significantly more frequent earplug use per week or day, while no association was found with earbud volume levels. Logistic regression revealed a strong association between earplug sensitization and the diagnosis of OE (odds ratio: 24.890, 95% CI: 1.945-318.481).

The higher reaction rate to Galaxy Buds Live may be due to its material composition, increased usage, and individual susceptibility. OE patients used in-ear devices more frequently (median 10 vs 21 times/week, *P* = .023), suggesting prolonged exposure raises sensitization risk. With the highest patch test positivity (8.0%), Galaxy Buds Live may contain stronger sensitizers, warranting further research.

This study has limitations, including a small sample size and reliance on self-reported data, which may introduce recall bias. Higher Galaxy Buds Live usage may confound the observed dermatitis incidence. Additionally, undiagnosed skin conditions, untested materials, and the lack of a direct correlation between patch test results and earplug use limit the generalizability of our findings. Although earbud use during exercise or water exposure was not specifically assessed, the tested devices lack IP5/8 or IP6/8 waterproof ratings and are not resistant to sweat or splashes according to their manuals, suggesting a consistent risk of moisture exposure across participants.

Contact allergy to earphones/earplugs was more common in OE patients, suggesting allergic contact dermatitis may contribute to the condition and should be considered in clinical evaluation.

## Conflicts of interest

None disclosed.
